# Transition Metal Complexes with Flufenamic Acid for Pharmaceutical Applications—A Novel Three-Centered Coordination Polymer of Mn(II) Flufenamate

**DOI:** 10.3390/ma13173705

**Published:** 2020-08-21

**Authors:** Michał Gacki, Karolina Kafarska, Anna Pietrzak, Małgorzata Szczesio, Izabela Korona-Głowniak, Wojciech M. Wolf

**Affiliations:** 1 Institute of General and Ecological Chemistry, Faculty of Chemistry, Lodz University of Technology, 116 Zeromskiego Street, 90–924 Lodz, Poland; karolina.kafarska@p.lodz.pl (K.K.); anna.pietrzak.1@p.lodz.pl (A.P.); malgorzata.szczesio@p.lodz.pl (M.S.); wojciech.wolf@p.lodz.pl (W.M.W.); 2 Department of Pharmaceutical Microbiology, Medical University of Lublin, Chodzki 1, 20–093 Lublin, Poland; iza.glowniak@umlub.pl

**Keywords:** non-steroidal anti-inflammatory drug-complex, coordination polymer, thermal analysis, antioxidant activity, flufenamic acid

## Abstract

Five complexes of Mn(II), Co(II), Ni(II), Cu(II) and Zn(II) with non-steroidal anti-inflammatory drug, flufenamic acid were synthesized: (1) [Mn_3_(fluf)_6_EtOH)(H_2_O)]_·_3EtOH; (2) [Co(fluf)_2_(EtOH)(H_2_O)]_·_H_2_O; (3) [Ni(fluf)_2_(EtOH)(H_2_O)]_·_H_2_O; (4) [Cu(fluf)_2·_H_2_O]; (5) [Zn(fluf)_2·_H_2_O]. All complexes were characterized by elemental analysis (EA), flame atomic absorption spectrometry (FAAS), Fourier-transform infrared spectroscopy (FTIR), and thermogravimetric analysis (TGA). The crystal structure of **1** was determined by the single crystal X-ray diffraction technique. It crystallizes in the triclinic space group P$\bar{1}$ with three independent Mn(II) cations, six coordinated flufenamato ligands augmented with water and ethanol molecules in the inner coordination sphere. In this crystal, manganese atoms are multiplied by symmetry and form infinite, polymeric chains which extend along the [001] dimension. The Hirshfeld Surface analysis revealed changes in interaction assemblies around all metal centers. The antioxidant and antimicrobial activities were established for all complexes and free ligand for comparison. All compounds exhibit good or moderate bioactivity against *Gram-positive bacteria* and yeasts.

## 1. Introduction

Coordination polymers (CPs) have attracted attention of numerous scientists, and material engineers well over 30 years [1]. The oldest known CP is the Prussian blue (Fe_4_[Fe(CN)_6_]3H_2_O) which, since the pioneering work of Keggin and Miles [2], initiated remarkable development in the field [3,4]. Following the IUPAC recommendation [5], CP is an array of constitution repeating units linked by coordinate–covalent bonding. Therefore, a one-dimensional coordination polymer contains metal ions or their clusters connected by coordinating organic ligands into an infinitive chain [6]. Progress in the efficient synthesis and design of CPs [7,8] prompted diverse applications in technologies crucial for civilization development. The gas and energy storage [9,10], intelligent drug delivery [11], production of smart catalysts and recent, remarkable advancements in tunable catalysis are among the most important of these applications [12,13].

**Scheme 1:**
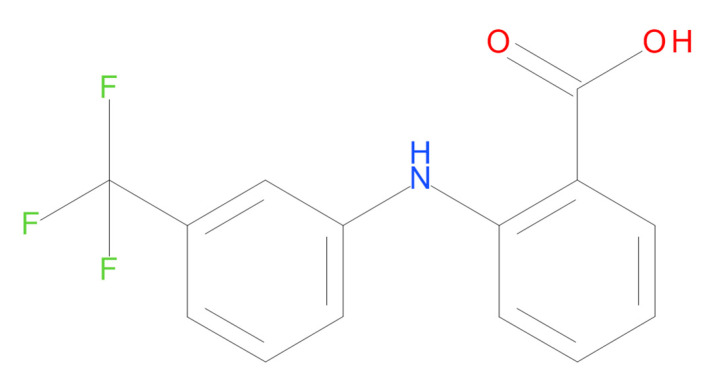
Structural scheme of flufenamic acid **6**.

Flufenamic acid (HFluf, Scheme 1.) 2–[3–(trifluoromethyl)phenyl] aminobenzoic acid is a member of anthranilic acid derivatives family (fenamates) [14]. It belongs to a group of non-steroidal anti-inflammatory drugs (NSAIDs). Their activity is based on the inhibition of cyclooxygenases COX–1 and COX–2 which hamper conversion of the arachidonic acid to prostaglandins and are responsible for anti-inflammatory action. Side effects are related to the well-known ability of COX–1 to prompt gastric irritations [15,16,17]. Hfluf has been used as analgesic, anti-inflammatory or antipyretic agent, applied in treatments of diverse rheumatic disorders, soft tissue injuries [18], musculoskeletal and joint disorders [18,19,20]. Further studies revealed its role as an ion channel modulator [20,21]. Especially, Hfluf interacts with either a non-selective cation and chloride channels or selective potassium, calcium and sodium channels [20]. Regrettably, HFluf, like other nonselective NSAIDs, exhibits side effects, namely gastrointestinal perturbations and renal damage [20,22]. In order to reduce those effects investigations in the field are directed towards the identification and design of selective COX–2 inhibitors. Heavy metal complexes with existing, non-specific drugs are also considered. It is well recognized that coordination of metal ions with pharmaceutically active substances may improve their activities and profoundly moderate toxicities [23,24]. While numerous metal–NSAIDS complexes were synthesized and thoroughly analyzed [25,26,27,28,29], only a few species with flufenamic acid have been reported, namely cobalt [30] nickel [19], copper [31,32,33,34,35,36] and zinc [14,36,37,38] complexes.

In this contribution, we report synthesis, spectroscopic characterization and thermal behavior of Mn, Co, Ni, Cu and Zn complexes with HFluf **1**–**5**, respectively. Antioxidant and antimicrobial activities of all investigated compounds augmented with those of free flufenamic acid were also determined. Moreover, the structure of **1** was solved and unequivocally refined by X-ray crystallography.

## 2. Results and Discussion

Compounds **1**–**5** are air stable at room temperature. They are highly soluble in polar organic solvents like methanol, ethanol DMF and DMSO. Their properties were thoroughly investigated by the elemental analysis (EA), flame atomic absorption spectrometry (FAAS), Fourier-transform infrared spectroscopy (FTIR) and thermogravimetric (TGA) methods augmented by antimicrobial and antioxidant studies, Table 1. Crystal structure of **1** was determined by the single crystal X-ray diffraction technique, Table 2.

### 2.1. Crystal Structure

#### 2.1.1. Description of Crystal Structure **1**

**Figure 1:**
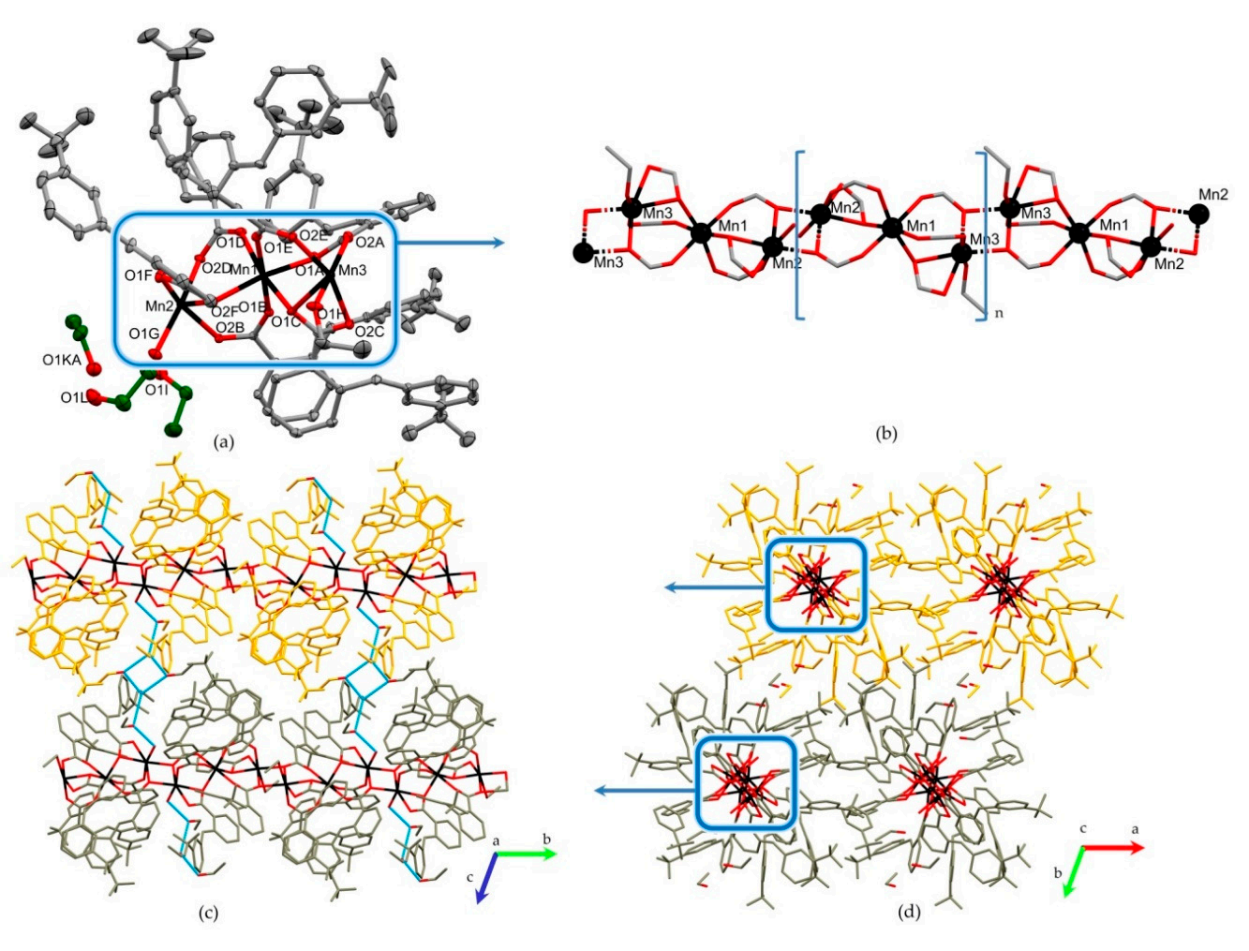
(**a**) Asymmetric unit of **1.** Manganese atoms are shown in black, oxygens are in red, ligands in the inner coordination sphere are in gray while non coordinating ethanol molecules are in green; (**b**) The coordination polymer chain. The constitutional repeating unit is shown in brackets; (**c**,**d**) Packing diagrams along [100] and [001], respectively. The coordination polymer chain is in black and red, while hydrogen bonding involving ethanol molecules is in blue.

Complex **1** crystallizes in the centrosymmetric, triclinic space group P$\bar{1}$ with asymmetric unit containing three independent Mn(II) cations, six coordinating flufenamato ligands augmented with water and ethanol molecules in the inner coordination sphere. Additionally, three disordered ethanol molecules are placed in voids out of the manganese coordination spheres (Figure 1a). All metal ions are six coordinated and exhibit twisted octahedral geometry. Deprotonated HFluf ligands (Fluf) link manganese cations by complexation with carboxylate groups. Two of them adopt a bidentate (µ_2_:η^1^:η^1^) binding mode while the remaining four ligands are tridentate (µ_2_:η^2^:η^1^). In particular, the central Mn1 ion is surrounded by six single oxygen O1A, O1B, O1C, O1D, O1E, O2F atoms from six diverse symmetrically independent carboxylate groups. Oxygens O1B and O1E occupy axial positions while O1A, O1C, O1D, O2F are equatorial. Parent oxygens of Fluf residues are also involved in coordination with the Mn2 and Mn3 wing atoms. Namely, O2B, O2D, O2F coordinate Mn2 while O2A, O1C, O2E are associated with Mn3. Oxygens O1F and O2C are exclusively connected to Mn2 and Mn3. Furthermore, coordination spheres of the latter ions are completed by O1G water and O1H ethanol molecules, respectively. Atoms O1C, O2B, O2E and O2F are bridging between neighboring Mn1–Mn2, Mn2–Mn2[1–x, 1–y, –z], Mn3–Mn3[1–x, 1–y, 1–z] and Mn1–Mn3 metal centers, respectively. In the crystal, those coordination bonds are multiplied by symmetry and form infinite, polymeric ribbons which expand along the [001] dimension (Figure 1b–d). Distances between the central Mn1 and adjacent Mn2, Mn3 ions are almost identical [Mn1^…^Mn2 and Mn1^…^Mn3 are 3.52, 3.57 Å, respectively] while contacts between wing manganese atoms are shorter, Mn2^…^Mn2 [1–x, 1–y, –z] and Mn3^…^Mn3 [1–x, 1–y, 1–z] are 3.25 and 3.24 Å, accordingly. The non-bonding pivotal angle Mn2^…^Mn1^…^Mn3 is 152.94°. The equatorial Mn–O bond lengths are within the range [2.1216(18)–2.1893(19) Å] while those in axial positions are slightly longer [2.1852(16)–2.2920(17) Å]. Relevant bond lengths and angles are summarized in Tables S1–S3.

Crystal is stabilized by an extensive network of inter- and intramolecular hydrogen bonds. Especially, strong intramolecular interactions are formed between amine groups (N1A, N1B, N1C, N1D, N1E and N1F) and the oxygen (O1A, O1B, O2C, O1D, O1E and O1F) atoms of carboxylate group of flufenamato ligands, Table 3. Hydrogen of the coordinated water molecule participates in the hydrogen bond O1G–H1G^…^O1I with the hydroxyl group of the neighboring ethanol molecule which is also involved in O1I–H1I^…^O1KA bonding with the consecutive ethanol molecule. The hydrogen H1KA of the latter species forms O1KA–H1KA^…^O1L bond with the third disordered ethanol molecule which is located in the void. Those interactions are replicated by the crystal symmetry and form chain $C_{6}^{5}\left(13\right)$ [39,40] which extends along the [010] dimension. The O1K and O1L ethanols are associated through the O1L–H1L^…^O1KA hydrogen bond which is transformed by the inversion center to form $R_{4}^{4}$ ring additionally stabilized by the chain O1KA–H1KA^…^O1L interaction, (Figure 1c).

#### 2.1.2. Hirshfeld Surface Analysis of the Mn^2+^ Centers

**Figure 2:**
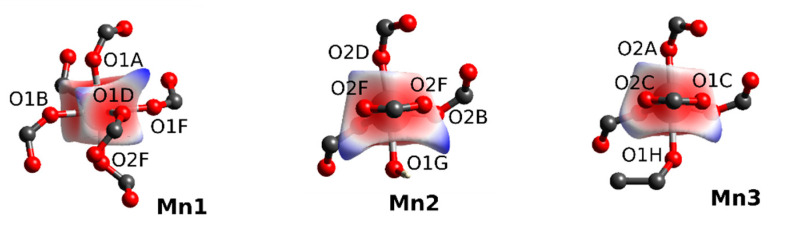
The Hirshfeld Surface of Mn^2+^ centers in **1** with a normalized contact distance (d_norm_) mapped on it. The parameter d_norm_ ranges from –0.76 to 0.76.

The structural investigations of **1** have been augmented by the Hirshfeld Surface (HS) analysis (University of Western Australia, Crawley, Australia) of symmetrically independent metal centers Mn1, Mn2 and Mn3. This approach has been successfully applied for metal complexes by Pinto et al. [41] and Abendrot et al. [42]. As shown in the Figure 2, HS of the central Mn1 is diverse than those generated for wing atoms Mn2 and Mn3. Their shapes reflect the differences in coordination of all Mn ions.

**Figure 3:**
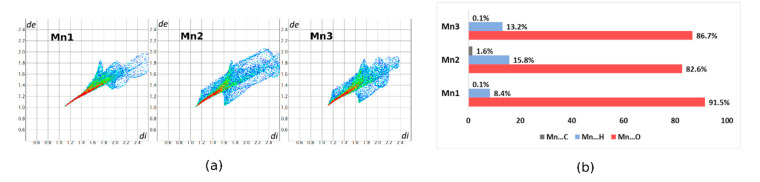
(**a**) Fingerprint plot for the Hirshfeld Surface of the metal centers Mn1, Mn2 and Mn3 of **1**; (**b**) The percentage contribution of contacts of the Mn^2+^ in the coordination polymer of **1**.

Figure 3 presents the Fingerprint Plots (FPs) for the HS of all respective metal centers. The central sharp streaks display the shortest Mn^…^O contacts (mainly red and green dots starting at about 1Å *d_i_* vs. *d_e_* scattergrams). Such plots also reflect subtle changes in interaction assembly around a metal center. Wing atoms Mn2 and Mn3 reveal the resemblance in the HS and FP shapes; however, the percentage contributions of Mn^…^O contacts is 4.1% higher for Mn3, suggesting that ethanol molecule may provide stronger Mn–O coordination than a sole water molecule. The coordination completed only by the carboxylate groups results in the highest contribution of Mn^…^O contacts (91.5%) to the HS of the central atom. Consequently, with increasing contributions of Mn^…^O contacts, we observe diminishing contributions of the scattered spots following the Mn^…^H interactions (13.2%, 15.8% and 8.4% for Mn3, Mn2 and Mn1, respectively). Moreover, a breakdown of FP’s for the particular metal center into individual contacts revealed the presence of Mn^…^C contacts in Mn2 at the level of 1.6%, while in Mn1 and Mn3 the contributions of similar contacts are negligible (0.1%). This weak (*d_i_* ≈ 1.8–2.4 Å and *d_e_* ≈ 1.8–2.0 Å) contact follows the formation of intramolecular contact between Mn2 and C2B of the central phenyl ring in the Fluf molecule.

### 2.2. Near Infrared Spectra of ***1***–***6***

**Figure 4:**
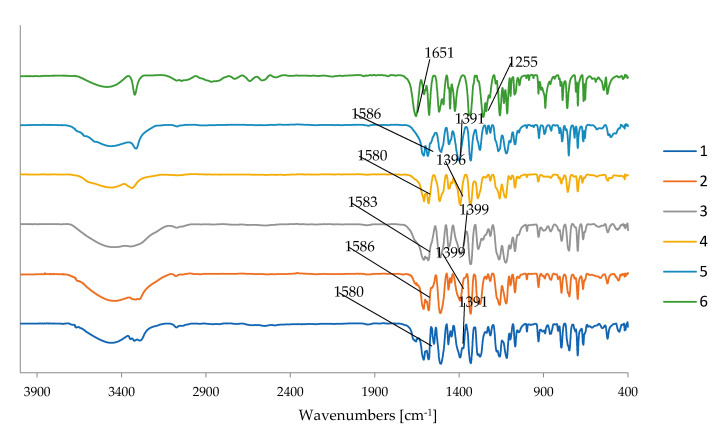
FTIR spectra of **1**–**5** complexes and free ligand **6**.

The infrared spectra of **1**–**6** were collected within the range 4000 and 400 cm^−1^ and are shown in the Figure 4. All complexes exhibit a broad absorption band 3300–3600 cm^−1^ which is assigned to the *ν*(OH) stretching vibrations of either water or ethanol molecules. Another appearance in this region were bands from *ν*(NH) stretching vibrations of flufenamato ligands.

The characteristic bands at 1651 and 1255 cm^−1^ are attributed to the respective *ν*(C=O) and *ν*(C–O) stretching vibrations of the carboxylic group in the free Hfluf. In **1**–**5**, they are shifted towards ranges 1586–1580 and 1399–1391 cm^−1^ which correspond to *ν*_as_(COO^−^) and *ν*_s_(COO^−^) stretching vibrations, respectively. Both are affected by ligands coordination to metal ions. Additionally, in the IR spectra of **1**–**5** there exist 460–490 cm^−1^ bands associated with metal-oxygen vibrations (Figures S1–S6). The parameter ∆*ν*(CO_2_) = *ν*_as_ – *ν*_s_ is slightly smaller for all complexes than that for sodium fenamate (∆*ν*(CO_2_) = 192 cm^−1^) [14] and, according to the well-recognized Nakamoto [43] criteria, the coordination mode of carboxylate groups is the bidentate chelating.

### 2.3. TG–DTG–DTA Anaylysis

**Figure 5:**
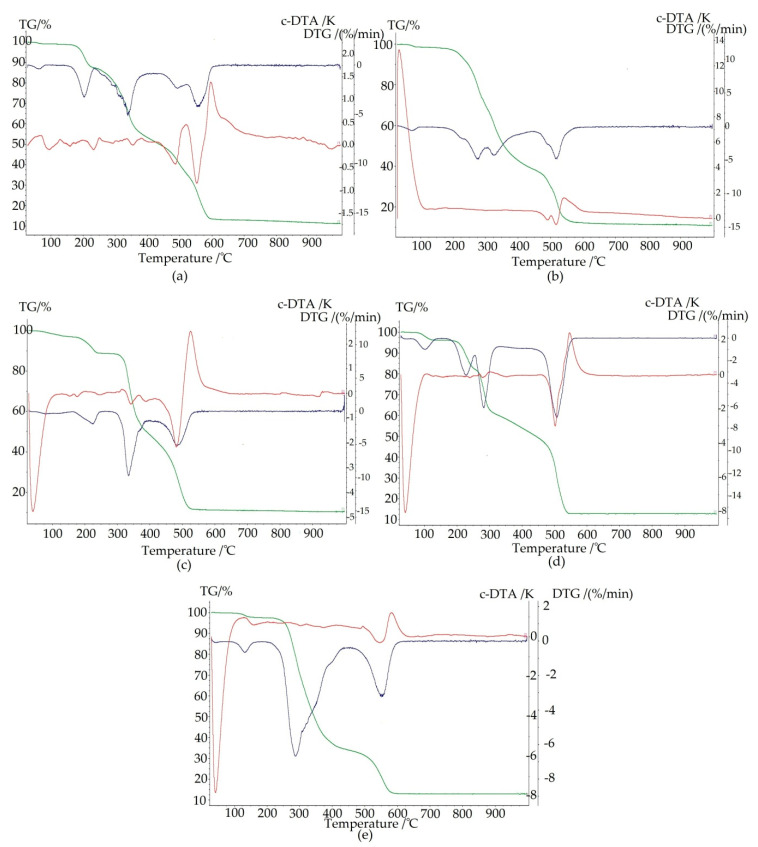
Thermoanalytical curves for **1** (**a**), **2** (**b**), **3** (**c**), **4** (**d**) and **5** (**e**). TG is plotted in green, DTG is in blue, while DTA is in red.

Figure 5 shows the TG/DTG/DTA curves of **1**–**5**, indicating that their decompositions are multistage processes. Compounds **2**–**5** start to decompose through the dehydration process at the 70–170 °C temperature range. It is associated with the loss of a sole water molecule as in **2**, **3**, **4** and **5** (mass loss exp. 2.12%, 2.72%, 3.70%, 2.30% and calc. 2.57%, 2.57%, 2.81%, 2.80%, respectively). The corresponding exo-effects were identified on respective DTA curves. Complex **1** begins to decompose by elimination of water together with all ethanol molecules (exp. 10.57% and calc. 9.88%) at 120–220 °C. It is accompanied by the endo- and egzo-thermic effects on the DTA curve. The following step in **3** (250–400 °C) corresponds to elimination one water and a single ethanol molecules (exp. 8.66% and calc. 9.15%). In **2**, it is additionally combined (200–400 °C) with the release of a half-flufenamate ligand (exp. 28.27% and calc. 29.12%). The characteristic exo- and endothermic effects related to the latter transformations are clearly visible on DTA curves. The second step of **1** and **4** decomposition (220–600 and 150–550 °C) is assigned to a ligand destruction in a single process (exp. 78.30%, calc. 78.82% and exp. 83.56%, calc. 84.80%), respectively. On the contrary, a two-step flufenamato elimination (250–600 °C) in **5** was observed (exp. 64.26%, calc. 64.28% and exp. 20.61%, calc. 20.28%). Further heating leads to the completely flufenamato ligand degradation in both **2** and **3**. The corresponding exo- and endothermal effects are visible on relevant DTA curves. The following mass losses were noted; exp. 58.75%, calc. 57.63% (400–600 °C); exp. 77.95%, calc. 77.63% (380–550 °C). The final products of thermal decompositions are Mn_3_O_4_ for **1**, CoO for **2**, NiO for **3**, CuO for **4** and ZnO for **5**. Their experimental residual masses (Mn_3_O_4_: 11.13%; CoO: 10.86%; NiO: 10.67%; CuO: 12.74%; ZnO: 12.83%) were close to calculated values (Mn_3_O_4_: 11.30%; CoO: 10.68%; NiO: 10.65%; CuO: 12.39%; ZnO: 12.64%).

### 2.4. Antioxidant and Antimicrobial Activities

The antibacterial, antifungal and antioxidant activities have been determined for all complexes **1**–**5** and additionally for the free Hfluf **6** which was taken as a reference. The in vitro antimicrobial activity has been evaluated against several bacterial and fungal reference strains (Table 4). Vancomycin (Van), ciprofloxacin (Cip) and nystatin (Nys) were used as the standard drugs. In this study, minimum inhibitory concentrations (MIC s) < 1000 mg/L were considered as noteworthy [44]. No bioactivity of tested compounds was observed for Gram-negative reference strains (MIC > 1000 mg/L) except for *P. mirabilis* strain which had inhibited growth in concentration of 250 mg/L for all tested compounds. From those findings, it was revealed that good bioactivity of tested compounds (MIC 26–125 mg/L) against most of Gram-positive reference bacteria was comparable to that of free ligand and was not affected by the coordinated metal ions. All compounds showed moderate activity (MIC 126–500 mg/L) against *S. mutans* and *E. faecalis* strains. Either free ligand or complexes demonstrated comparable, moderate antifungal activity against *Candida* spp. reference strains.

The DPPH radical scavenging activities (RSA) are low with the exception of **4** which exhibits noticeable RSA (60.72%).

## 3. Experimental

### 3.1. Preparation of Complexes and Crystallization

Compounds **1**–**5** were synthesized by the following procedure. Flufenamic acid (2 mmol) was dissolved in 30 mL of a freshly prepared aqueous-ethanol solution (*v*/*v* 1:2) of NaOH (0.02 mol·L^−1^). Then, sodium flufenamate was heated up to 60 °C and subsequently added to an aqueous-ethanol solution (*v*/*v* 1:2) of respective metal chlorides (1.0 mol in 15 mL) heated to the same temperature. Reaction mixtures were carried out at 50 °C for 3 h. After a few days, the polycrystalline powders of **2**–**5** were isolated by filtration, washed with a hot water, and dried in air. Green single crystals of **1** suitable for X-ray structure analysis were obtained from mother liquor after one week. Numerous attempts to obtain single crystals of **2**–**5** were unsuccessful.

### 3.2. Materials and Methods

Flufenamic acid was purchased from Sigma-Aldrich (Poznań, Poland) and used without further purification. Ethanol and DPPH (2,2–diphenyl–1–picrylhydrazyl) were purchased from POCH, Gliwice, Poland. All reagents were chemically pure.

The chemical compositions of **1**–**5** were determined by the elemental analysis and flame atomic absorption spectrometry (FAAS) (Complexes (20 mg) were mineralized by the Anton Paar Multiwave 3000 closed microwave system (Anton Paar GmbH, Graz, Austria); the mixture of concentrated HNO_3_ and HCl (6:1, *v*/*v*) was applied. Metals concentration were measured by the HR–CS FAAS with the ContrAA 300 Analytik Jena spectrometer (Analytik Jena, Jena, Germany) plus spectrometer. Hydrogen, carbon and nitrogen contents were measured with the Vario EL III Elemental Analyzer (Elementar Analysensysteme GmbH, Langenselold, Germany).

Infrared spectra of **1**–**6** were recorded with the Thermo Scientific Nicolet 6700 FTIR spectrometer (Thermo Scientific, Waltham, MA, USA) equipped with a liquid nitrogen-cooled MCT (Mercury cadmium telluride—MCT, HgCdTe) detector (Thermo Scientific, Waltham, MA, USA). Samples were prepared as KBr pellets and measured over the range 4000–400 cm^−1^.

Thermal stability and decomposition pathways of **1**–**5** were studied by thermogravimetric techniques. All complexes were measured with the Netzsch TG 209 apparatus (Netzsch, Selb, Germany). Samples (10 mg) were heated up to 1000 °C, at a heating rate 10 °C min^−1^ in air atmosphere.

X-ray data were collected at 100 K on the XtaLAB Synergy, Dualflex, Pilatus 300K diffractometer apparatus (Rigaku Corporation, Tokyo, Japan) equipped with the PhotonJet microfocus X-ray tube apparatus (Rigaku Corporation, Tokyo, Japan). Data reduction was performed with CrysAlisPro (Agilent Technologies UK Ltd., Yarnton, England) [45]. The structure was solved by Intrinsic Phasing as implemented in ShelXT [46] and further refined on F^2^ in ShelXL [47] by full-matrix least-squares minimalization. Non-hydrogen atoms were refined anisotropically. Four of six trifluoromethyl groups are rotationally disordered. Sites of disordered hydrogen atoms were identified as peaks on difference Fourier map and divided into groups using PART instruction. Their occupancy ratios were determined by the refinement of respective free variables. Geometrical parameters of disordered –CF_3_ groups were constrained to geometrically and spatially reasonable values using DFIX, DANG and SAME procedures. Moreover, atomic displacement ellipsoids of disordered fluorine atoms were restrained using SIMU, DELU and RIGU procedures. Anisotropic displacement parameters of fluorine atoms in molecule A were constrained in pairs by the EADP procedure. Hydrogen atoms were placed in calculated positions and refined riding on their parent atoms. The complex **1** crystallizes with three ethanol molecules placed in voids out of the Mn coordination sphere. The residual electron density map suggested substantial ethanol deficiency in the crystal. Freely refined occupancies of all three ethanol molecules add up to approximately 2.5 molecules per asymmetric unit. To validate that non-stoichometric number, the structure was subjected to the PLATON/SQUEEZE [48] procedure. The total potential solvent accessible void volume was ca. 577.7 Å^3^, the electron count per unit cell was 118 which corresponds to 59 electrons per asymmetric unit. Bearing in mind that ethanol molecule has 26 electrons, the latter is equivalent to 2.3 ethanol species per asymmetric unit. This number is approximately equal to the sum of refined ethanol occupancies.

Molecular plots and packing diagrams were drawn using Mercury [49]. Molecular geometry parameters were computed with PLATON [50].

Hirshfeld Surfaces of metallic centers Mn1, Mn2 and Mn3 were generated using the CrystalExplorer 17.5 program (University of Western Australia, Crawley, Australia). Molecular geometries were the same as in crystal structures. Only major components of crystallographic disorders were considered. On the generated HSs of Mn^2+^ centers, the normalized contact distances (d_norm_) were mapped. The explored d_norm_ function is defined as the sum of the distances from the HS to the nearest atom interior and exterior to the surface (d_i_ and d_e_, respectively). Distances (d_i_ and d_e_) were calculated and plotted as scattergrams, namely Fingerprint Plots (FPs) [51,52,53,54]. A quantitative decomposition analysis of atom to surface contacts was calculated as a percentage of the points in the Hirshfeld Surface with d_i_ and d_e_ for specific atom pairs.

The CIF file for **1** is available from the Cambridge Crystallographic Data Centre (CCDC) (deposition numbers CCDC: 2019658).

The antibacterial and antifungal activities were screened for **1**–**6** via the micro-dilution broth method according to the European Committee on Antimicrobial Susceptibility Testing (EUCAST) (www.eucast.org). Minimal inhibitory concentrations (MIC) of the tested compounds were evaluated for the panel of the reference microorganisms from the American Type Culture Collection (ATCC), including Gram-negative bacteria (*Salmonella Typhimurium* ATCC14028, *Escherichia coli* ATCC 25922, *Proteus mirabilis* ATCC 12453, *Klebsiella pneumoniae* ATCC 13883 and *Pseudomonas aeruginosa* ATCC 9027), Gram-positive bacteria (*Staphylococcus aureus* ATCC 25923, *Staphylococcus epidermidis* ATCC 12228, *Micrococcus luteus* ATCC 10240, *Enterococcus faecalis* ATCC 29212 *Bacillus subtilis* ATCC 6633, *Bacillus cereus* ATCC 10876*, Streptococcus pyogenes* ATCC 19615, *Streptococcus pneumoniae* ATCC 49619, and *Streptococcus mutans* ATCC 25175), and fungi (*Candida albicans* ATCC 10231, *Candida parapsilosis* ATCC 22019, and *Candida glabrata* ATCC 90030).

The antibacterial activities of **1**–**6** and their radical scavenging activity (RSA) were measured by method as described by us in detail [28].

## 4. Conclusions

In this contribution we synthesized and thoroughly investigated metal [Mn(II), Co(II), Ni(II), Cu(II) and Zn(II)] complexes with flufenamic acid. The structure of [Mn_3_(fluf)_6_(H_2_O)(EtOH)]·3EtOH was determined by X-ray crystallography. Asymmetric unit of that crystal contains three independent Mn(II) cations, six coordinated flufenamato ligands augmented with water and ethanol molecules in the inner coordination sphere. Additionally, three disordered ethanol molecules are placed in voids. Flufenamato ligands are bound to manganese ions by carboxylato groups which adopt distinct binding modes. Manganese atoms form infinite, polymeric 1-D chains which extend along the [001] dimension. Their Hirshfeld Surface analysis highlighted reduction of Mn^…^O contacts contribution within coordination spheres prompted by replacement of carboxylate ligands with water and ethanol as in Mn2 and Mn3, respectively. This study firmly showed that geometrical properties mapped on the HS of the metal centers in crystals may be a useful tool to identify subtle effects in the metal coordination spheres.

The investigated compounds exhibited moderate to good activities against Gram-positive bacteria and yeasts. Moreover, the Cu(II) complex is a good antioxidant scavenger as determined in the experiment with the DPPH free radical.

## Figures and Tables

**Table 1:** Analytical data for **1**–**5**.

No.	Compounds	Empirical Formula	Color	Formula Weight [g/mol]	Elemental Analysis, Found (calc.) [%]			
					C	H	N	M *
**1**	[Mn_3_(fluf)_6_(H_2_O)(EtOH)]·3EtOH	C_92_H_80_F_18_Mn_3_N_6_O_17_	Pale pink	2048.44	54.21 (53.94)	3.87 (3.94)	4.67 (4.10)	8.36 (8.05)
**2**	[Co(fluf)_2_(EtOH)(H_2_O)]·H_2_O	C_30_H_28_F_6_CoN_2_O_7_	Pink	701.48	51.68 (51.37)	3.51 (4.02)	3.77 (3.99)	8.78 (8.40)
**3**	[Ni(fluf)_2_(EtOH)(H_2_O)]·H_2_O	C_30_H_28_F_6_NiN_2_O_7_	Green	701.24	51.88 (51.38)	3.61 (4.02)	3.67 (3.99)	8.18 (8.37)
**4**	[Cu(fluf)_2_H_2_O]	C_28_H_20_F_6_CuN_2_O_5_	Green	642.02	52.21 (52.38)	3.87 (3.14)	4.27 (4.36)	9.71 (9.90)
**5**	[Zn(fluf)_2_(H_2_O)]	C_28_H_20_F_6_ZnN_2_O_5_	White	643.86	52.21 (52.23)	3.02 (3.13)	4.17 (4.35)	10.18 (10.15)

* M represents metal content by FAAS spectroscopy.

**Table 2:** Crystal data and structure refinement parameters for **1**.

	1
T (K)	100.0
Crystal system	triclinic
Space group	P$\bar{1}$
a (Å)	15.2886 (3)
b (Å)	17.4001 (4)
c (Å)	18.0882 (4)
α (°)	98.841 (2)
β (°)	93.192 (2)
γ (°)	108.922 (2)
*V* (Å^3^)	4469.01 (18)
Z	2
*ρ*_calc_ (g/cm^3^)	1.495
F(000)	2052
Radiation	MoKα (λ = 0.71073)
2*θ* range (°)	3.469–31.506
Reflections collected	70043
Independent reflections	23878
Goodness–of–fit (GOF)	1.073
R [I ≥ 2σ (I)]	0.0539
*w*R_2_ [I ≥ 2σ (I)]	0.1289

**Table 3:** Hydrogen bond geometry in **1** (Å,°).

D–H^…^A	D–H	H^…^A	D^…^A	D–H^…^A
N1A–H1A^…^O1A	0.80 (4)	2.00 (4)	2.642 (3)	137 (3)
N1B–H1B^…^O1B	0.84 (3)	2.03 (3)	2.686 (3)	134 (3)
N1C–H1C^…^O2C	0.88 (3)	1.93 (3)	2.643 (3)	138 (3)
N1D–H1D^…^O1D	0.83 (3)	2.02 (3)	2.671 (3)	134 (3)
N1E–H1E^…^O1E	0.86 (3)	2.10 (3)	2.718 (3)	129 (3)
N1F–H1F^…^O1F	0.88 (3)	1.92 (4)	2.638 (3)	138 (4)
O1G–H1G^…^O1I	0.68 (3)	1.99 (3)	2.636 (3)	160 (4)
O1I–H1I^…^O1KA	0.74 (5)	2.02 (5)	2.751 (4)	170 (4)
O1KA–H1KA^…^O1L	0.82	1.85	2.665 (5)	173
O1L–H1L…O1KA^a^	0.82	2.03	2.824 (5)	163

Symmetry codes: (^a^) 1–x, 1–y, –z.

**Table 4:** The antibacterial and antifungal activities.

Chemicals Microorganism	1	2	3	4	5	6	Van/Nys
	MIC [mg/L]						
***Gram-Positive bacteria***							**Van**
*S. aureus* ATCC 25923	62.5	62.5	62.5	62.5	62.5	62.5	0.98
*S. epidermidis* ATCC 12228	62.5	62.5	62.5	62.5	62.5	62.5	0.98
*M. luteus* ATCC 10240	125	62.5	62.5	62.5	31.3	62.5	0.12
*E. faecalis* ATCC 29212	500	500	500	500	500	500	1.95
*B. subtilis* ATCC 6633	62.5	62.5	62.5	62.5	62.5	62.5	0.24
*B. cereus* ATCC 10876	62.5	31.3	62.5	62.5	62.5	31.3	0.98
*S. pyogenes* ATCC 19615	125	125	125	125	125	125	0.24
*S. pneumoniae* ATCC 49619	62.5	62.5	125	125	125	125	0.24
*S. mutans* ATCC 25175	250	250	250	250	250	125	0.98
***Gram-Negative bacteria***							**Cip**
*S. typhimurium* ATCC 14028	>1000	>1000	>1000	>1000	>1000	>1000	0.061
*E. coli* ATCC 25922	>1000	>1000	>1000	>1000	>1000	>1000	0.015
*P. mirabilis* ATCC 12453	250	250	250	250	250	250	0.030
*K. pneumoniae* ATCC 13883	>1000	>1000	>1000	>1000	>1000	>1000	0.122
*P. aeruginosa* ATCC 9027	>1000	>1000	>1000	>1000	>1000	>1000	0.488
**Yeasts**							**Nys**
*C. albicans* ATCC 2091	250	250	250	250	250	250	0.48
*C. parapsilosis* ATCC 22019	250	250	250	250	250	250	0.24
*C. glabrata* ATCC 90030	500	250	500	500	500	500	0.24

## Supplementary Materials

The following are available online at https://www.mdpi.com/1996-1944/13/17/3705/s1, Figure S1: title, Table S1: title, Video S1: The supplementary material includes: Near–IR spectra for **1**–**6** (Tables S1–S6, respectively). Bond distances (Table S1), bond angles (Table S2) and torsion angles (Table S3) for **1**.
