# Supplementary material for: Transition Metal Complexes with Flufenamic Acid for Pharmaceutical Applications—A Novel Three-Centered Coordination Polymer of Mn(II) Flufenamate
Source: Materials (Basel). 2020 Aug 21;13(17):3705. doi: 10.3390/ma13173705 (PMC7503579; doi:10.3390/ma13173705)
Supplement: Supplementary file 1 [file materials-13-03705-s001.pdf]

Supporting information

Transition Metal Complexes with Flufenamic Acid for Pharmaceutical Applications—A novel Three-Centered Coordination Polymer of Mn(II) Flufenamate

Michał Gacki [1,*], Karolina Kafarska [1], Anna Pietrzak [1], Małgorzata Szczesio [1], Izabela Korona-Głowniak [2] and Wojciech M. Wolf [1]

[1] Institute of General and Ecological Chemistry, Faculty of Chemistry, Lodz University of Technology, 116 Zeromskiego Street, 90–924 Lodz, Poland; karolina.kafarska@p.lodz.pl (K.K.); anna.pietrzak.1@p.lodz.pl (A.P.); malgorzata.szczesio@p.lodz.pl (M.S.); wojciech.wolf@p.lodz.pl (W.M.W.)

[2] Department of Pharmaceutical Microbiology, Medical University of Lublin, Chodzki 1, 20–093 Lublin, Poland; iza.glowniak@umlub.pl

* Correspondence: michal.gacki@edu.p.lodz.pl

Received: 30 July 2020; Accepted: 19 August 2020; Published: 21 August 2020

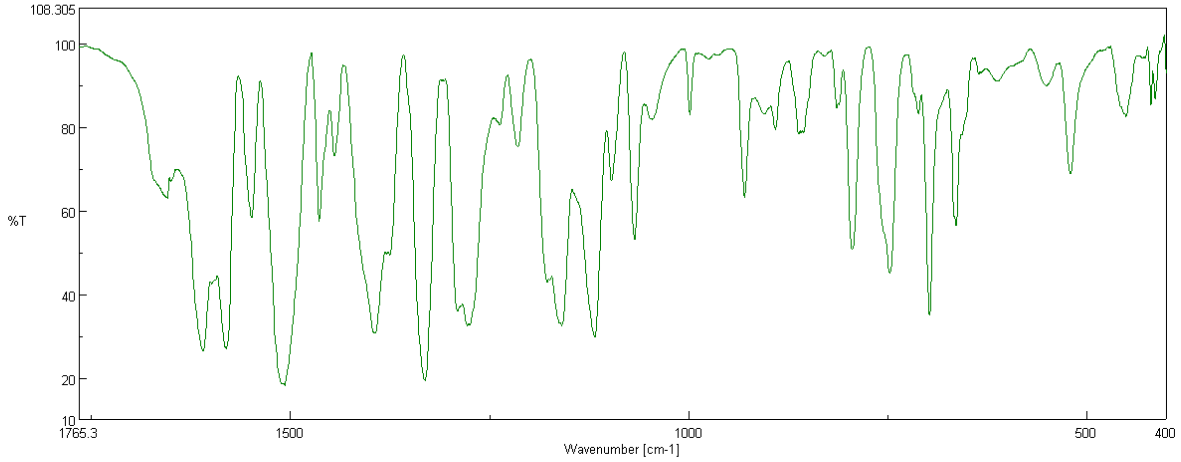

Figure S1. Near-IR spectra for 1.

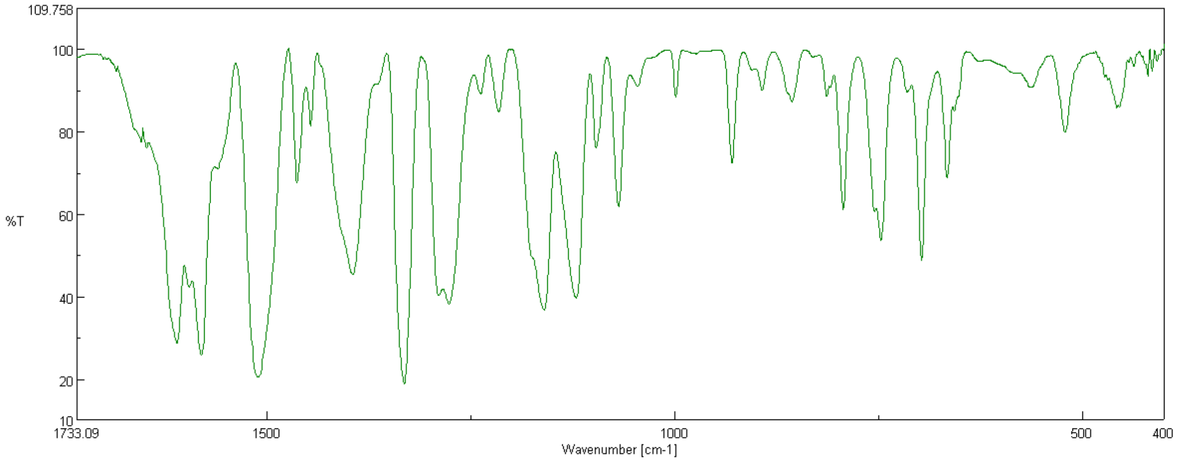

Figure S2. Near-IR spectra for 2.

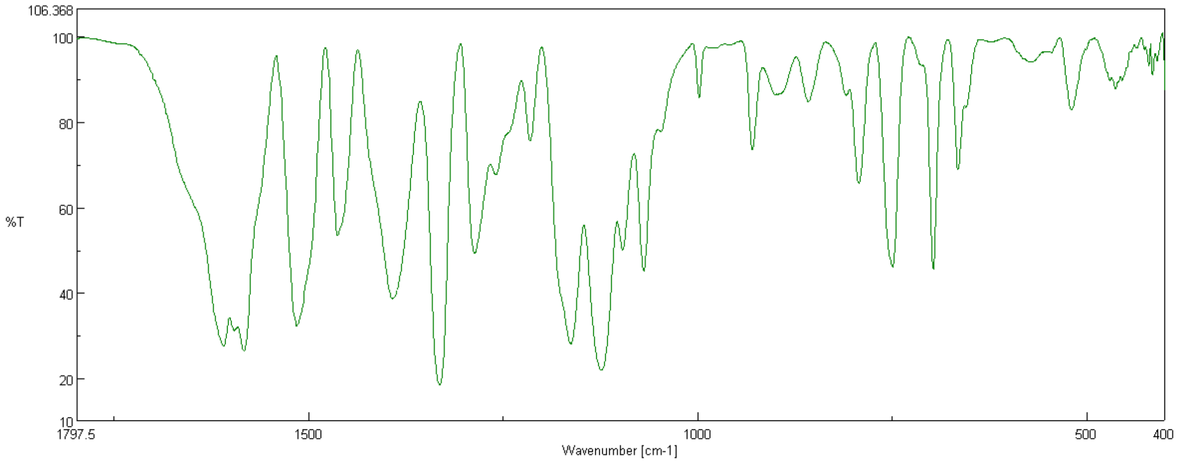

Figure S3. Near-IR spectra for 3.

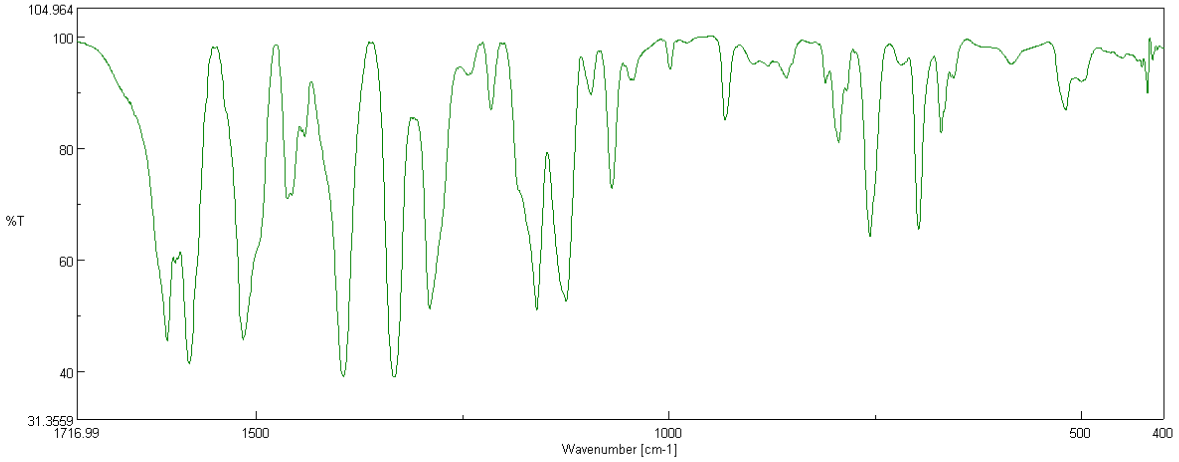

Figure S4. Near-IR spectra for 4.

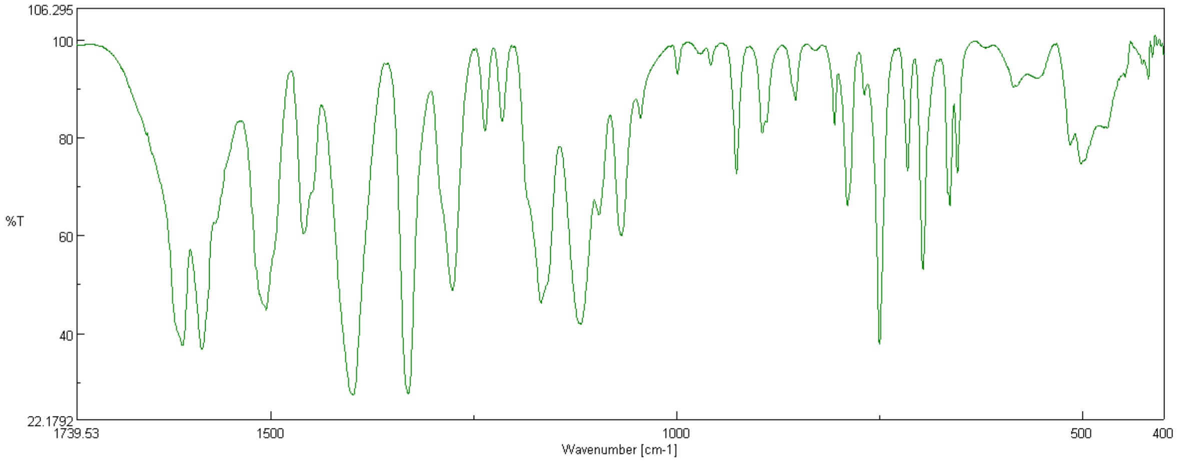

Figure S5. Near-IR spectra for 5.

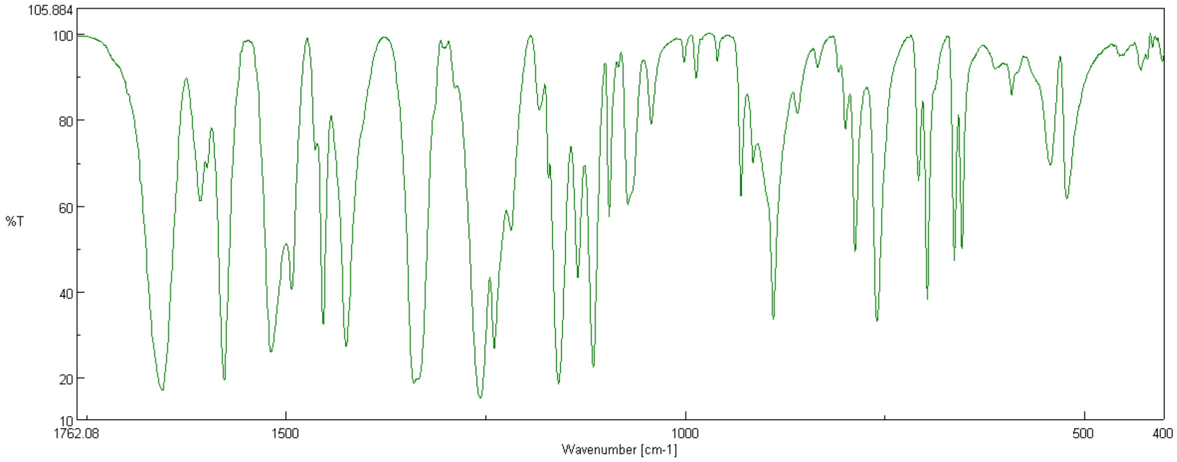

Figure S6. Near-IR spectra for 6.

Table S1. Bond distances for 1 (Å).

Mn1-O1A	2.1445(17)	F2F-C14F	1.342(5)
Mn1-O1B	2.1875(17)	F2G-C1G	1.319(19)
Mn1-O1C	2.1847(18)	F3E_2 -C14E_2	1.289(10)
Mn1-O1D	2.1724(18)	F3A-C14A	1.357(6)
Mn1-O1E	2.1922(17)	F3B-C14B	1.36(2)
Mn1-O2F	2.1435(17)	F3C-C14C	1.341(4)
Mn2-O1F	2.1893(19)	F3F-C14F	1.333(4)
Mn2-O1G	2.169(2)	F3G-C1G	1.32(2)
Mn2-O2B	2.1639(17)	F4E_2 -C14E_2	1.282(11)
Mn2-O2D	2.1216(18)	F4A-C14A	1.24(2)
Mn2-O2F	2.2920(17)	F4B-C14	1.33(4)
Mn2-O2B_a	2.2091(16)	F4G-C1G	1.320(14)
Mn3-O1C	2.2761(17)	F5E_2 -C14E_2	1.354(9)
Mn3-O1H	2.215(2)	F5A-C14A	1.29(3)
Mn3-O2A	2.1594(18)	F5B-C14	1.27(3)
Mn3-O2C	2.1726(18)	F5G-C1G	1.321(18)
Mn3-O2E	2.1450(18)	F6E_2 -C14E_2	1.295(10)
Mn3-O2E_b	2.1852(16)	F6A-C14A	1.49(3)
F1E_2 -C14E_2	1.258(11)	F6B-C14	1.32(3)
F1A-C14A	1.346(6)	F6G-C1G	1.343(13)
F1B-C14B	1.334(19)	O1A-C1A	1.266(3)
F1C-C14C	1.342(4)	O1B-C1B	1.255(3)
F1F-C14F	1.332(4)	O1C-C1C	1.284(3)
F1G-C1G	1.32(2)	O1D-C1D	1.266(3)
F2E_2 -C14E_2	1.363(7)	O1E-C1E	1.250(3)
F2A-C14A	1.304(5)	O1F-C1F	1.265(3)
F2B-C14B	1.39(2)	O1H-C1H	1.444(4)
F2C-C14C	1.351(4)	O2A-C1A	1.271(3)
O2B-C1B	1.285(3)	N1D-H1D	0.83(3)
O2C-C1C	1.273(3)	N1E-H1E	0.86(3)
O2D-C1D	1.270(3)	C1E-C2E	1.492(3)
O2E-C1E	1.287(3)	C1F-C2F	1.476(3)
O2F-C1F	1.289(3)	N1F-H1F	0.88(3)
N1A-C7A	1.385(3)	C1G-C10D	1.501(4)
N1A-C8A	1.397(4)	C1H-C2H	1.503(5)

N1B-C7B	1.380(3)	C2A-C7A	1.411(3)
N1B-C8B	1.412(3)	C2A-C3A	1.402(3)
N1C-C7C	1.389(3)	C2B-C3B	1.402(3)
N1C-C8C	1.396(3)	C2B-C7B	1.421(3)
N1D-C7D	1.374(3)	C2C-C7C	1.415(3)
N1D-C8D	1.411(3)	C2C-C3C	1.406(3)
N1E-C7E	1.381(3)	C2D-C7D	1.418(3)
N1E-C8E	1.407(3)	C2D-C3D	1.402(3)
N1F-C7F	1.379(3)	C2E-C7E	1.416(3)
N1F-C8F	1.402(4)	C2E-C3E	1.399(3)
O1G-H1G	0.68(3)	C2F-C3F	1.404(4)
O1H-H1H	0.86(3)	C2F-C7F	1.422(3)
C14E_2 -C10E	1.495(4)	C3A-C4A	1.383(4)
O1KA -C1K	1.414(6)	C3B-C4B	1.379(4)
N1A-H1A	0.80(4)	C3C-C4C	1.376(4)
C1A-C2A	1.498(3)	C3D-C4D	1.382(4)
N1B-H1B	0.84(3)	C3E-C4E	1.381(4)
C1B-C2B	1.491(3)	C3F-C4F	1.377(4)
C1C-C2C	1.473(3)	C4A-C5A	1.391(4)
N1C-H1C	0.88(3)	C4B-C5B	1.393(4)
C1D-C2D	1.490(4)	C4C-C5C	1.395(4)
C4D-C5D	1.391(4)	C9B-C10B	1.392(4)
C4E-C5E	1.396(4)	C9C-C10C	1.396(4)
C4F-C5F	1.398(4)	C9D-C10D	1.390(4)
C5A-C6A	1.377(4)	C9E-C10E	1.388(4)
C5B-C6B	1.377(4)	C9F-C10F	1.388(4)
C5C-C6C	1.379(4)	C10A -C14A	1.493(6)
C5D-C6D	1.378(4)	C10A -C11A	1.378(4)
C5E-C6E	1.372(4)	C10B -C14B	1.488(19)
C5F-C6F	1.381(4)	C10B -C11B	1.379(4)
C6A-C7A	1.408(3)	C10B -C14	1.528(18)
C6B-C7B	1.409(4)	C10C -C14C	1.486(4)
C6C-C7C	1.407(3)	C10C -C11C	1.383(4)
C6D-C7D	1.413(4)	C10D -C11D	1.381(4)
C6E-C7E	1.411(4)	C10E -C11E	1.390(4)
C6F-C7F	1.407(3)	C10F -C11F	1.383(5)
C8A-C13A	1.392(4)	C10F -C14F	1.494(5)
C8A-C9A	1.397(4)	C11A -C12A	1.393(6)
C8B-C13B	1.393(4)	C11B -C12B	1.379(4)
C8B-C9B	1.389(4)	C11C -C12C	1.388(4)
C8C-C9C	1.391(4)	C11D -C12D	1.388(4)
C8C-C13C	1.402(4)	C11E -C12E	1.381(4)
C8D-C9D	1.390(4)	C11F -C12F	1.385(5)
C8D-C13D	1.399(4)	C12A -C13A	1.389(5)
C8E-C13E	1.396(4)	C12B -C13B	1.392(4)
C8E-C9E	1.395(4)	C12C -C13C	1.385(4)
C8F-C9F	1.392(4)	C12D -C13D	1.382(4)
C8F-C13F	1.395(4)	C12E -C13E	1.391(4)
C9A-C10A	1.393(4)	C12F -C13F	1.392(4)
O1KA -H1KA	0.8200	C6E-H6E	0.9300
C1H-H1HB	0.9700	C6F-H6F	0.9300
C1H-H1HA	0.9700	C9A-H9A	0.9300

C2H-H2HB	0.9600	C9B-H9B	0.9300
C2H-H2HA	0.9600	C9C-H9C	0.9300
C2H-H2HC	0.9600	C9D-H9D	0.9300
C3A-H3A	0.9300	C9E-H9E	0.9300
C3B-H3B	0.9300	C9F-H9F	0.9300
C3C-H3C	0.9300	C11A -H11A	0.9300
C3D-H3D	0.9300	C11B -H11B	0.9300
C3E-H3E	0.9300	C11C -H11C	0.9300
C3F-H3F	0.9300	C11D -H11D	0.9300
C4A-H4A	0.9300	C11E -H11E	0.9300
C4B-H4B	0.9300	C11F -H11F	0.9300
C4C-H4C	0.9300	C12A -H12A	0.9300
C4D-H4D	0.9300	C12B -H12B	0.9300
C4E-H4E	0.9300	C12C -H12C	0.9300
C4F-H4F	0.9300	C12D -H12D	0.9300
C5A-H5A	0.9300	C12E -H12E	0.9300
C5B-H5B	0.9300	C12F -H12F	0.9300
C5C-H5C	0.9300	C13A -H13A	0.9300
C5D-H5D	0.9300	C13B -H13B	0.9300
C5E-H5E	0.9300	C13C -H13C	0.9300
C5F-H5F	0.9300	C13D -H13D	0.9300
C6A-H6A	0.9300	C13E -H13E	0.9300
C6B-H6B	0.9300	C13F -H13F	0.9300
C6C-H6C	0.9300	O1I-C1I	1.422(5)
C6D-H6D	0.9300	C1K-C2K	1.461(8)
O1I-H1I	0.74(5)	O1L-H1L	0.8200
C1K-H1K2	0.9700	C2I-H2IB	0.9600
C1K-H1K1	0.9700	C2I-H2IA	0.9600
C2K-H2KB	0.9600	C2I-H2IC	0.9600
C2K-H2KA	0.9600	C1L-C2L	1.492(8)
C2K-H2KC	0.9600	C1L-H1LA	0.9700
C1I-C2I	1.482(7)	C1L-H1LB	0.9700
O1L-C1L	1.411(7)	C2L-H2LA	0.9600
C1I-H1IA	0.9700	C2L-H2LB	0.9600
C1I-H1IB	0.9700	C2L-H2LC	0.9600

Table S2. Bond angles for 1 (°).

O1A-Mn1-O1B	82.67(6)	O2B_a-Mn2-O2D	85.16(6)
O1A-Mn1-O1C	86.04(7)	O2B_a-Mn2-O2F	172.21(6)
O1A-Mn1-O1D	87.79(7)	O1C-Mn3-O1H	87.25(7)
O1A-Mn1-O1E	96.82(6)	O1C-Mn3-O2A	102.37(6)
O1A-Mn1-O2F	170.85(7)	O1C-Mn3-O2C	58.98(6)
O1B-Mn1-O1C	94.93(6)	O1C-Mn3-O2E	100.54(6)
O1B-Mn1-O1D	93.90(7)	O1C-Mn3-O2E_b	169.88(7)
O1B-Mn1-O1E	176.39(7)	O1H-Mn3-O2A	169.63(7)
O1B-Mn1-O2F	89.83(6)	O1H-Mn3-O2C	90.14(7)
O1C-Mn1-O1D	168.50(6)	O1H-Mn3-O2E	81.94(7)
O1C-Mn1-O1E	88.60(6)	O1H-Mn3-O2E_b	83.86(7)
O1C-Mn1-O2F	89.40(6)	O2A-Mn3-O2C	98.12(7)
O1D-Mn1-O1E	82.50(7)	O2A-Mn3-O2E	92.36(7)
O1D-Mn1-O2F	98.00(7)	O2A-Mn3-O2E_b	86.86(6)
O1E-Mn1-O2F	90.99(6)	O2C-Mn3-O2E	158.54(6)

O1F-Mn2-O1G	91.04(7)	O2C-Mn3-O2E_b	116.08(6)
O1F-Mn2-O2B	154.59(7)	O2E-Mn3-O2E_b	83.02(6)
O1F-Mn2-O2D	97.65(7)	Mn1-O1A-C1A	138.52(16)
O1F-Mn2-O2F	58.59(6)	Mn1-O1B-C1B	145.28(15)
O1F-Mn2-O2B_a	120.91(7)	Mn1-O1C-Mn3	106.41(7)
O1G-Mn2-O2B	86.69(7)	Mn1-O1C-C1C	129.78(15)
O1G-Mn2-O2D	166.40(7)	Mn3-O1C-C1C	88.95(13)
O1G-Mn2-O2F	90.82(7)	Mn1-O1D-C1D	132.13(16)
O1G-Mn2-O2B_a	81.39(7)	Mn1-O1E-C1E	147.91(16)
O2B-Mn2-O2D	89.83(7)	Mn2-O1F-C1F	93.39(14)
O2B-Mn2-O2F	96.11(6)	Mn3-O1H-C1H	129.15(18)
O2B-Mn2-O2B_a	83.79(6)	Mn3-O2A-C1A	123.94(15)
O2D-Mn2-O2F	102.63(6)	Mn2-O2B-C1B	124.71(15)
Mn2-O2B-Mn2_a	96.21(7)	F3E_2-C14E_2 -C10E	112.3(5)
Mn2_a-O2B-C1B	129.85(14)	F4E_2-C14E_2 -F5E_2	100.6(9)
Mn3-O2C-C1C	93.97(14)	F6E_2-C14E_2 -C10E	114.4(5)
Mn2-O2D-C1D	132.82(15)	C8A-N1A-H1A	116(3)
Mn3-O2E-C1E	122.35(14)	C7A-N1A-H1A	116(3)
Mn3-O2E-Mn3_b	96.98(7)	O1A-C1A-C2A	118.0(2)
Mn3_b-O2E-C1E	136.07(16)	O2A-C1A-C2A	118.8(2)
Mn1-O2F-Mn2	105.20(7)	O1A-C1A-O2A	123.1(2)
Mn1-O2F-C1F	126.28(15)	O2B-C1B-C2B	117.05(19)
Mn2-O2F-C1F	88.11(13)	C8B-N1B-H1B	117(2)
C7A-N1A-C8A	127.2(2)	C7B-N1B-H1B	116(2)
C7B-N1B-C8B	126.3(2)	O1B-C1B-C2B	120.33(19)
C7C-N1C-C8C	129.4(2)	O1B-C1B-O2B	122.6(2)
C7D-N1D-C8D	125.2(2)	O2C-C1C-C2C	121.7(2)
C7E-N1E-C8E	124.2(2)	O1C-C1C-C2C	120.27(19)
C7F-N1F-C8F	129.5(2)	O1C-C1C-O2C	118.0(2)
Mn2-O1G-H1G	109(3)	C8C-N1C-H1C	115(2)
C1H-O1H-H1H	107.2(17)	C7C-N1C-H1C	114(2)
Mn3-O1H-H1H	118.1(17)	O2D-C1D-C2D	117.1(2)
F1E_2-C14E_2 -F2E_2	107.4(7)	C8D-N1D-H1D	118(2)
F1E_2-C14E_2 -F3E_2	110.3(7)	O1D-C1D-C2D	119.7(2)
F1E_2-C14E_2 -C10E	116.1(5)	O1D-C1D-O2D	123.2(2)
F2E_2-C14E_2 -F3E_2	100.4(6)	C7D-N1D-H1D	117(2)
F4E_2-C14E_2 -F6E_2	110.8(7)	O1E-C1E-C2E	120.3(2)
F4E_2-C14E_2 -C10E	116.6(6)	O2E-C1E-C2E	117.04(19)
F5E_2-C14E_2 -F6E_2	103.6(9)	C8E-N1E-H1E	115(2)
F5E_2-C14E_2 -C10E	109.1(6)	O1E-C1E-O2E	122.7(2)
F2E_2-C14E_2 -C10E	109.1(4)	C7E-N1E-H1E	120(2)
O1F-C1F-O2F	118.5(2)	C1D-C2D-C7D	123.7(2)
C7F-N1F-H1F	115(3)	C3D-C2D-C7D	118.9(2)
C8F-N1F-H1F	116(3)	C3E-C2E-C7E	119.2(2)
O1F-C1F-C2F	121.7(2)	C1E-C2E-C3E	118.5(2)
O2F-C1F-C2F	119.8(2)	C1E-C2E-C7E	122.3(2)
F4G-C1G-F5G	108.3(10)	C1F-C2F-C3F	117.9(2)
F4G-C1G-F6G	107.0(10)	C1F-C2F-C7F	122.4(2)
F4G-C1G-C10D	112.2(7)	C3F-C2F-C7F	119.7(2)
F5G-C1G-F6G	104.0(13)	C2A-C3A-C4A	121.4(2)
F5G-C1G-C10D	113.6(8)	C2B-C3B-C4B	122.4(2)
F6G-C1G-C10D	111.4(6)	C2C-C3C-C4C	120.7(2)

F1G-C1G-F2G	107.8(19)	C2D-C3D-C4D	122.2(3)
F1G-C1G-F3G	103.7(16)	C2E-C3E-C4E	121.9(2)
F1G-C1G-C10D	113.1(13)	C2F-C3F-C4F	121.6(2)
F2G-C1G-F3G	106.3(18)	C3A-C4A-C5A	119.1(3)
F2G-C1G-C10D	111.4(12)	C3B-C4B-C5B	118.6(2)
F3G-C1G-C10D	114.0(12)	C3C-C4C-C5C	119.5(2)
O1H-C1H-C2H	111.7(3)	C3D-C4D-C5D	118.8(3)
C3A-C2A-C7A	119.2(2)	C3E-C4E-C5E	118.6(2)
C1A-C2A-C3A	118.3(2)	C3F-C4F-C5F	118.5(3)
C1A-C2A-C7A	122.4(2)	C4A-C5A-C6A	120.7(3)
C3B-C2B-C7B	118.8(2)	C4B-C5B-C6B	120.6(3)
C1B-C2B-C3B	118.3(2)	C4C-C5C-C6C	120.9(2)
C1B-C2B-C7B	122.9(2)	C4D-C5D-C6D	120.7(3)
C1C-C2C-C3C	117.6(2)	C4E-C5E-C6E	120.8(2)
C1C-C2C-C7C	122.5(2)	C4F-C5F-C6F	121.5(2)
C3C-C2C-C7C	119.9(2)	C5A-C6A-C7A	121.0(2)
C1D-C2D-C3D	117.5(2)	C5B-C6B-C7B	121.7(2)
C5C-C6C-C7C	120.7(2)	N1C-C8C-C9C	124.2(2)
C5D-C6D-C7D	121.4(3)	N1C-C8C-C13C	116.6(2)
C5E-C6E-C7E	121.3(2)	C9C-C8C-C13C	119.0(2)
C5F-C6F-C7F	120.8(2)	N1D-C8D-C13D	121.1(2)
N1A-C7A-C6A	120.1(2)	N1D-C8D-C9D	119.6(2)
C2A-C7A-C6A	118.5(2)	C9D-C8D-C13D	119.4(2)
N1A-C7A-C2A	121.4(2)	N1E-C8E-C13E	119.4(2)
N1B-C7B-C2B	120.7(2)	N1E-C8E-C9E	121.3(2)
C2B-C7B-C6B	117.8(2)	C9E-C8E-C13E	119.3(2)
N1B-C7B-C6B	121.4(2)	C9F-C8F-C13F	118.9(3)
N1C-C7C-C2C	119.6(2)	N1F-C8F-C9F	116.8(2)
N1C-C7C-C6C	122.0(2)	N1F-C8F-C13F	124.1(3)
C2C-C7C-C6C	118.3(2)	C8A-C9A-C10A	119.9(3)
N1D-C7D-C2D	121.0(2)	C8B-C9B-C10B	119.6(2)
N1D-C7D-C6D	121.0(2)	C8C-C9C-C10C	119.4(2)
C2D-C7D-C6D	118.0(2)	C8D-C9D-C10D	119.5(3)
N1E-C7E-C2E	122.1(2)	C8E-C9E-C10E	120.0(2)
N1E-C7E-C6E	119.9(2)	C8F-C9F-C10F	120.5(3)
C2E-C7E-C6E	118.0(2)	C9A-C10A -C11A	121.1(3)
N1F-C7F-C2F	119.5(2)	C9A-C10A -C14A	118.7(3)
N1F-C7F-C6F	122.6(2)	C11A -C10A -C14A	120.2(3)
C2F-C7F-C6F	117.9(2)	C9B-C10B -C11B	121.2(3)
N1A-C8A-C9A	122.5(2)	C9B-C10B -C14	117.9(8)
N1A-C8A-C13A	118.4(3)	C9B-C10B -C14B	121.2(8)
C9A-C8A-C13A	118.9(3)	C11B -C10B -C14	120.5(8)
N1B-C8B-C9B	121.7(2)	C11B -C10B -C14B	117.4(8)
N1B-C8B-C13B	118.9(2)	C9C-C10C -C11C	121.6(3)
C9B-C8B-C13B	119.4(2)	C9C-C10C -C14C	119.3(3)
C11C -C10C -C14C	119.0(3)	F4B-C14-F5B	111(2)
C1G-C10D -C11D	120.1(3)	F4B-C14-F6B	106.9(19)
C9D-C10D -C11D	121.3(3)	F4B-C14-C10B	114.4(19)
C1G-C10D -C9D	118.5(3)	F5B-C14-F6B	103(2)
C9E-C10E -C11E	120.7(3)	F5B-C14-C10B	112.4(16)
C14E_2 -C10E -C9E	118.4(3)	F6B-C14-C10B	109.2(19)
C14E_2 -C10E -C11E	120.8(3)	F3A-C14A -C10A	111.6(3)

C9F-C10F -C11F	120.8(3)	F1A-C14A -F2A	108.0(4)
C11F -C10F -C14F	121.8(3)	F1A-C14A -F3A	106.1(4)
C9F-C10F -C14F	117.4(3)	F1A-C14A -C10A	111.7(3)
C10A -C11A -C12A	119.1(3)	F2A-C14A -F3A	106.4(5)
C10B -C11B -C12B	119.2(3)	F2A-C14A -C10A	112.7(4)
C10C -C11C -C12C	118.9(3)	F4A-C14A -C10A	117.6(11)
C10D -C11D -C12D	119.0(3)	F4A-C14A -F5A	111.1(17)
C10E -C11E -C12E	119.2(3)	F4A-C14A -F6A	100.7(15)
C10F -C11F -C12F	118.7(3)	F6A-C14A -C10A	109.1(10)
C11A -C12A -C13A	120.3(3)	F5A-C14A -F6A	95.2(15)
C11B -C12B -C13B	120.6(2)	F5A-C14A -C10A	118.9(14)
C11C -C12C -C13C	120.4(3)	F3B-C14B -C10B	114.4(13)
C11D -C12D -C13D	120.5(3)	F2B-C14B -C10B	112.2(14)
C11E -C12E -C13E	120.8(3)	F1B-C14B -F2B	103.1(14)
C11F -C12F -C13F	121.2(3)	F1B-C14B -F3B	103.8(14)
C8A-C13A -C12A	120.6(3)	F1B-C14B -C10B	111.0(12)
C8B-C13B -C12B	120.1(2)	F2B-C14B -F3B	111.4(14)
C8C-C13C -C12C	120.7(3)	F2C-C14C -F3C	105.7(2)
C8D-C13D -C12D	120.3(2)	F2C-C14C -C10C	112.1(3)
C8E-C13E -C12E	120.0(3)	F1C-C14C -C10C	113.0(2)
C8F-C13F -C12F	119.8(3)	F1C-C14C -F2C	106.0(3)
F1C-C14C -F3C	106.6(2)	C2E-C3E-H3E	119.00
F3C-C14C -C10C	112.9(3)	C4E-C3E-H3E	119.00
F3F-C14F -C10F	112.5(3)	C2F-C3F-H3F	119.00
F1F-C14F -F2F	106.8(3)	C4F-C3F-H3F	119.00
F1F-C14F -F3F	106.1(3)	C5A-C4A-H4A	120.00
F1F-C14F -C10F	113.4(3)	C3A-C4A-H4A	120.00
F2F-C14F -F3F	105.3(3)	C3B-C4B-H4B	121.00
F2F-C14F -C10F	112.3(3)	C5B-C4B-H4B	121.00
C1K-O1KA -H1KA	109.00	C5C-C4C-H4C	120.00
O1H-C1H-H1HB	109.00	C3C-C4C-H4C	120.00
C2H-C1H-H1HA	109.00	C3D-C4D-H4D	121.00
C2H-C1H-H1HB	109.00	C5D-C4D-H4D	121.00
H1HA -C1H-H1HB	108.00	C5E-C4E-H4E	121.00
O1H-C1H-H1HA	109.00	C3E-C4E-H4E	121.00
C1H-C2H-H2HA	110.00	C3F-C4F-H4F	121.00
C1H-C2H-H2HB	110.00	C5F-C4F-H4F	121.00
H2HA -C2H-H2HB	110.00	C6A-C5A-H5A	120.00
H2HA -C2H-H2HC	109.00	C4A-C5A-H5A	120.00
H2HB -C2H-H2HC	109.00	C4B-C5B-H5B	120.00
C1H-C2H-H2HC	109.00	C6B-C5B-H5B	120.00
C4A-C3A-H3A	119.00	C6C-C5C-H5C	120.00
C2A-C3A-H3A	119.00	C4C-C5C-H5C	120.00
C2B-C3B-H3B	119.00	C4D-C5D-H5D	120.00
C4B-C3B-H3B	119.00	C6D-C5D-H5D	120.00
C4C-C3C-H3C	120.00	C4E-C5E-H5E	120.00
C2C-C3C-H3C	120.00	C6E-C5E-H5E	120.00
C4D-C3D-H3D	119.00	C6F-C5F-H5F	119.00
C2D-C3D-H3D	119.00	C4F-C5F-H5F	119.00
C7A-C6A-H6A	120.00	C10C -C11C -H11C	121.00
C5A-C6A-H6A	119.00	C12C -C11C -H11C	121.00
C7B-C6B-H6B	119.00	C12D -C11D -H11D	120.00

C5B-C6B-H6B	119.00	C10D -C11D -H11D	121.00
C5C-C6C-H6C	120.00	C12E -C11E -H11E	120.00
C7C-C6C-H6C	120.00	C10E -C11E -H11E	120.00
C5D-C6D-H6D	119.00	C10F -C11F -H11F	121.00
C7D-C6D-H6D	119.00	C12F -C11F -H11F	121.00
C7E-C6E-H6E	119.00	C13A -C12A -H12A	120.00
C5E-C6E-H6E	119.00	C11A -C12A -H12A	120.00
C5F-C6F-H6F	120.00	C11B -C12B -H12B	120.00
C7F-C6F-H6F	120.00	C13B -C12B -H12B	120.00
C10A -C9A-H9A	120.00	C13C -C12C -H12C	120.00
C8A-C9A-H9A	120.00	C11C -C12C -H12C	120.00
C8B-C9B-H9B	120.00	C11D -C12D -H12D	120.00
C10B -C9B-H9B	120.00	C13D -C12D -H12D	120.00
C10C -C9C-H9C	120.00	C11E -C12E -H12E	120.00
C8C-C9C-H9C	120.00	C13E -C12E -H12E	120.00
C8D-C9D-H9D	120.00	C13F -C12F -H12F	119.00
C10D -C9D-H9D	120.00	C11F -C12F -H12F	119.00
C10E -C9E-H9E	120.00	C12A -C13A -H13A	120.00
C8E-C9E-H9E	120.00	C8A-C13A -H13A	120.00
C8F-C9F-H9F	120.00	C8B-C13B -H13B	120.00
C10F -C9F-H9F	120.00	C12B -C13B -H13B	120.00
C12A -C11A -H11A	120.00	C8C-C13C -H13C	120.00
C10A -C11A -H11A	120.00	C12C -C13C -H13C	120.00
C10B -C11B -H11B	120.00	C12D -C13D -H13D	120.00
C12B -C11B -H11B	120.00	C8D-C13D -H13D	120.00
C8E-C13E -H13E	120.00	C2I-C1I-H1IB	109.00
C12E -C13E -H13E	120.00	H1IA -C1I-H1IB	108.00
C12F -C13F -H13F	120.00	C1L-O1L-H1L	109.00
C8F-C13F -H13F	120.00	C1I-C2I-H2IB	110.00
O1KA -C1K-C2K	109.8(4)	C1I-C2I-H2IC	109.00
C1I-O1I-H1I	109(4)	H2IA -C2I-H2IB	109.00
O1KA -C1K-H1K1	110.00	H2IA -C2I-H2IC	109.00
O1KA -C1K-H1K2	110.00	H2IB -C2I-H2IC	109.00
C2K-C1K-H1K2	110.00	C1I-C2I-H2IA	110.00
H1K1 -C1K-H1K2	108.00	O1L-C1L-C2L	109.5(5)
C2K-C1K-H1K1	110.00	O1L-C1L-H1LA	110.00
C1K-C2K-H2KB	110.00	O1L-C1L-H1LB	110.00
C1K-C2K-H2KC	109.00	C2L-C1L-H1LA	110.00
H2KA -C2K-H2KB	109.00	C2L-C1L-H1LB	110.00
H2KA -C2K-H2KC	109.00	H1LA -C1L-H1LB	108.00
H2KB -C2K-H2KC	109.00	C1L-C2L-H2LA	109.00
C1K-C2K-H2KA	110.00	C1L-C2L-H2LB	109.00
O1I-C1I-C2I	113.6(3)	C1L-C2L-H2LC	110.00
O1I-C1I-H1IA	109.00	H2LA -C2L-H2LB	109.00
O1I-C1I-H1IB	109.00	H2LA -C2L-H2LC	109.00
C2I-C1I-H1IA	109.00	H2LB -C2L-H2LC	110.00

Table S7. Torsion angles for **1**(°).

O1B-Mn1-O1A-C1A	-163.2(3)
O1C-Mn1-O1A-C1A	-67.7(3)
O1D-Mn1-O1A-C1A	102.6(3)
O1E-Mn1-O1A-C1A	20.4(3)

O1A-Mn1-O1B-C1B	-175.9(3)
O1C-Mn1-O1B-C1B	98.7(3)
O1D-Mn1-O1B-C1B	-88.7(3)
O2F-Mn1-O1B-C1B	9.3(3)
O1A-Mn1-O1C-Mn3	53.48(7)
O1A-Mn1-O1C-C1C	-49.24(19)
O1B-Mn1-O1C-Mn3	135.76(7)
O1B-Mn1-O1C-C1C	33.04(19)
O1E-Mn1-O1C-Mn3	-43.46(7)
O1E-Mn1-O1C-C1C	-146.18(19)
O2F-Mn1-O1C-Mn3	-134.46(7)
O2F-Mn1-O1C-C1C	122.82(19)
O1A-Mn1-O1D-C1D	120.8(2)
O1B-Mn1-O1D-C1D	38.3(2)
O1E-Mn1-O1D-C1D	-142.0(2)
O2F-Mn1-O1D-C1D	-52.1(2)
O1A-Mn1-O1E-C1E	-82.6(3)
O1C-Mn1-O1E-C1E	3.3(3)
O1D-Mn1-O1E-C1E	-169.4(3)
O2F-Mn1-O1E-C1E	92.7(3)
O1B-Mn1-O2F-Mn2	-48.15(8)
O1B-Mn1-O2F-C1F	-147.18(18)
O1C-Mn1-O2F-Mn2	-143.08(7)
O1C-Mn1-O2F-C1F	117.89(18)
O1D-Mn1-O2F-Mn2	45.76(8)
O1D-Mn1-O2F-C1F	-53.27(19)
O1E-Mn1-O2F-Mn2	128.33(7)
O1E-Mn1-O2F-C1F	29.30(18)
O1G-Mn2-O1F-C1F	83.26(15)
O2B-Mn2-O1F-C1F	-1.2(2)
O2D-Mn2-O1F-C1F	-107.23(15)
O2F-Mn2-O1F-C1F	-7.07(13)
O2B_a-Mn2-O1F-C1F	163.84(13)
O1F-Mn2-O2B-C1B	-43.6(3)
O1F-Mn2-O2B-Mn2_a	167.15(13)
O1G-Mn2-O2B-C1B	-129.05(19)
O1G-Mn2-O2B-Mn2_a	81.69(7)
O2D-Mn2-O2B-C1B	64.11(18)
O2D-Mn2-O2B-Mn2_a	-85.15(7)
O2F-Mn2-O2B-C1B	-38.58(19)
O2F-Mn2-O2B-Mn2_a	172.16(6)
O2B_a-Mn2-O2B-C1B	149.26(19)
O2B_a-Mn2-O2B-Mn2_a	0.00(7)
O1F-Mn2-O2D-C1D	46.8(2)
O2B-Mn2-O2D-C1D	-108.9(2)
O2F-Mn2-O2D-C1D	-12.6(2)
O2B_a-Mn2-O2D-C1D	167.4(2)
O1F-Mn2-O2F-Mn1	-120.27(10)
O1F-Mn2-O2F-C1F	6.92(13)
O1G-Mn2-O2F-Mn1	149.00(8)
O1G-Mn2-O2F-C1F	-83.80(14)
O2B-Mn2-O2F-Mn1	62.25(8)

O2B-Mn2-O2F-C1F	-170.56(14)
O2D-Mn2-O2F-Mn1	-28.95(9)
O2D-Mn2-O2F-C1F	98.24(14)
O1F-Mn2-O2B_a-Mn2_a	-173.61(7)
O1F-Mn2-O2B_a-C1B_a	-26.8(2)
O1G-Mn2-O2B_a-Mn2_a	-87.57(8)
O1G-Mn2-O2B_a-C1B_a	59.2(2)
O2B-Mn2-O2B_a-Mn2_a	-0.02(10)
O2B-Mn2-O2B_a-C1B_a	146.8(2)
O2D-Mn2-O2B_a-Mn2_a	90.36(7)
O2D-Mn2-O2B_a-C1B_a	-122.8(2)
O1H-Mn3-O1C-Mn1	138.65(8)
O1H-Mn3-O1C-C1C	-89.93(14)
O2A-Mn3-O1C-Mn1	-37.43(8)
O2A-Mn3-O1C-C1C	94.00(14)
O2C-Mn3-O1C-Mn1	-129.54(10)
O2C-Mn3-O1C-C1C	1.89(13)
O2E-Mn3-O1C-Mn1	57.37(8)
O2E-Mn3-O1C-C1C	-171.20(13)
O1C-Mn3-O1H-C1H	68.6(2)
O2C-Mn3-O1H-C1H	9.7(2)
O2E-Mn3-O1H-C1H	169.7(2)
O2E_b-Mn3-O1H-C1H	-106.5(2)
O1C-Mn3-O2A-C1A	-11.2(2)
O2C-Mn3-O2A-C1A	48.72(19)
O2E-Mn3-O2A-C1A	-112.50(19)
O2E_b-Mn3-O2A-C1A	164.63(19)
O1C-Mn3-O2C-C1C	-1.91(13)
O1H-Mn3-O2C-C1C	84.79(15)
O2A-Mn3-O2C-C1C	-101.51(14)
O2E-Mn3-O2C-C1C	17.0(3)
O2E_b-Mn3-O2C-C1C	168.03(13)
O1C-Mn3-O2E-C1E	-30.12(18)
O1C-Mn3-O2E-Mn3_b	170.42(6)
O1H-Mn3-O2E-C1E	-115.78(18)
O1H-Mn3-O2E-Mn3_b	84.75(7)
O2A-Mn3-O2E-C1E	72.92(18)
O2A-Mn3-O2E-Mn3_b	-86.54(7)
O2C-Mn3-O2E-C1E	-46.5(3)
O2C-Mn3-O2E-Mn3_b	154.05(15)
O2E_b-Mn3-O2E-C1E	159.46(18)
O2E_b-Mn3-O2E-Mn3_b	-0.02(10)
O1H-Mn3-O2E_b-Mn3_b	-82.59(8)
O1H-Mn3-O2E_b-C1E_b	72.1(2)
O2A-Mn3-O2E_b-Mn3_b	92.77(7)
O2A-Mn3-O2E_b-C1E_b	-112.5(2)
O2C-Mn3-O2E_b-Mn3_b	-169.73(6)
O2C-Mn3-O2E_b-C1E_b	-15.0(2)
O2E-Mn3-O2E_b-Mn3_b	0.02(11)
O2E-Mn3-O2E_b-C1E_b	154.7(2)
Mn1-O1A-C1A-C2A	-160.01(18)
Mn1-O1A-C1A-O2A	22.5(4)

Mn1-O1B-C1B-C2B	-155.8(2)
Mn1-O1B-C1B-O2B	23.6(4)
Mn1-O1C-C1C-C2C	-74.8(3)
Mn1-O1C-C1C-O2C	107.5(2)
Mn3-O1C-C1C-O2C	-3.1(2)
Mn3-O1C-C1C-C2C	174.6(2)
Mn1-O1D-C1D-O2D	13.2(4)
Mn1-O1D-C1D-C2D	-168.12(16)
Mn1-O1E-C1E-O2E	33.0(4)
Mn1-O1E-C1E-C2E	-145.8(2)
Mn2-O1F-C1F-O2F	12.3(2)
Mn2-O1F-C1F-C2F	-165.1(2)
Mn3-O1H-C1H-C2H	67.2(3)
Mn3-O2A-C1A-O1A	26.2(3)
Mn3-O2A-C1A-C2A	-151.26(17)
Mn2_a-O2B-C1B-O1B	136.84(19)
Mn2-O2B-C1B-O1B	-1.7(3)
Mn2_a-O2B-C1B-C2B	-43.8(3)
Mn2-O2B-C1B-C2B	177.68(15)
Mn3-O2C-C1C-C2C	-174.4(2)
Mn3-O2C-C1C-O1C	3.3(2)
Mn2-O2D-C1D-C2D	-153.45(17)
Mn2-O2D-C1D-O1D	25.3(4)
Mn3_b-O2E-C1E-C2E	-42.6(3)
Mn3-O2E-C1E-O1E	-11.3(3)
Mn3_b-O2E-C1E-O1E	138.6(2)
Mn3-O2E-C1E-C2E	167.53(15)
Mn2-O2F-C1F-O1F	-11.7(2)
Mn1-O2F-C1F-C2F	-86.7(3)
Mn2-O2F-C1F-C2F	165.8(2)
Mn1-O2F-C1F-O1F	95.9(2)
C7A-N1A-C8A-C13A	155.8(3)
C7A-N1A-C8A-C9A	-29.3(4)
C8A-N1A-C7A-C6A	-29.6(4)
C8A-N1A-C7A-C2A	152.7(3)
C7B-N1B-C8B-C9B	47.8(4)
C7B-N1B-C8B-C13B	-134.1(3)
C8B-N1B-C7B-C6B	9.6(4)
C8B-N1B-C7B-C2B	-172.6(2)
C7C-N1C-C8C-C13C	-165.8(3)
C8C-N1C-C7C-C6C	29.7(4)
C7C-N1C-C8C-C9C	18.7(4)
C8C-N1C-C7C-C2C	-153.6(3)
C8D-N1D-C7D-C6D	12.0(4)
C7D-N1D-C8D-C9D	-129.4(3)
C8D-N1D-C7D-C2D	-170.3(2)
C7D-N1D-C8D-C13D	52.5(4)
C8E-N1E-C7E-C6E	30.0(4)
C7E-N1E-C8E-C9E	31.1(4)
C8E-N1E-C7E-C2E	-151.3(2)
C7E-N1E-C8E-C13E	-150.5(2)
C8F-N1F-C7F-C6F	-0.7(4)

C7F-N1F-C8F-C13F	-42.3(4)
C8F-N1F-C7F-C2F	-178.6(3)
C7F-N1F-C8F-C9F	142.7(3)
F3E_2-C14E_2 -C10E -C11E	37.3(7)
F3E_2-C14E_2 -C10E -C9E	-144.9(6)
F1E_2-C14E_2 -C10E -C11E	165.4(7)
F2E_2-C14E_2 -C10E -C9E	104.8(5)
F2E_2-C14E_2 -C10E -C11E	-73.1(5)
F1E_2-C14E_2 -C10E -C9E	-16.8(7)
O2A-C1A-C2A-C7A	-167.0(2)
O1A-C1A-C2A-C7A	15.4(4)
O1A-C1A-C2A-C3A	-160.5(2)
O2A-C1A-C2A-C3A	17.1(4)
O2B-C1B-C2B-C7B	164.1(2)
O1B-C1B-C2B-C7B	-16.5(3)
O1B-C1B-C2B-C3B	160.8(2)
O2B-C1B-C2B-C3B	-18.6(3)
O2C-C1C-C2C-C3C	165.3(2)
O1C-C1C-C2C-C3C	-12.4(3)
O1C-C1C-C2C-C7C	168.8(2)
O2C-C1C-C2C-C7C	-13.5(4)
O2D-C1D-C2D-C3D	4.1(3)
O1D-C1D-C2D-C7D	5.4(4)
O2D-C1D-C2D-C7D	-175.8(2)
O1D-C1D-C2D-C3D	-174.7(2)
O2E-C1E-C2E-C3E	-20.1(3)
O1E-C1E-C2E-C3E	158.8(2)
O1E-C1E-C2E-C7E	-19.3(3)
O2E-C1E-C2E-C7E	161.9(2)
O2F-C1F-C2F-C7F	172.9(2)
O2F-C1F-C2F-C3F	-8.0(3)
O1F-C1F-C2F-C3F	169.4(2)
O1F-C1F-C2F-C7F	-9.8(4)
F4G-C1G-C10D -C11D	-4.6(8)
F6G-C1G-C10D -C11D	-124.4(11)
F5G-C1G-C10D -C11D	118.6(9)
F5G-C1G-C10D -C9D	-64.0(9)
F4G-C1G-C10D -C9D	172.8(7)
F6G-C1G-C10D -C9D	53.0(12)
C1A-C2A-C3A-C4A	174.6(3)
C7A-C2A-C3A-C4A	-1.5(4)
C3A-C2A-C7A-N1A	179.5(3)
C1A-C2A-C7A-C6A	-174.1(2)
C1A-C2A-C7A-N1A	3.6(4)
C3A-C2A-C7A-C6A	1.8(4)
C1B-C2B-C7B-C6B	179.6(2)
C1B-C2B-C7B-N1B	1.7(4)
C3B-C2B-C7B-N1B	-175.6(2)
C7B-C2B-C3B-C4B	-1.4(4)
C1B-C2B-C3B-C4B	-178.9(2)
C3B-C2B-C7B-C6B	2.3(3)
C1C-C2C-C7C-C6C	179.6(2)

C3C-C2C-C7C-C6C	0.9(3)
C1C-C2C-C7C-N1C	2.8(4)
C7C-C2C-C3C-C4C	-0.2(4)
C3C-C2C-C7C-N1C	-176.0(2)
C1C-C2C-C3C-C4C	-179.1(2)
C3D-C2D-C7D-C6D	-2.0(4)
C1D-C2D-C7D-N1D	0.0(4)
C7D-C2D-C3D-C4D	0.5(4)
C1D-C2D-C3D-C4D	-179.3(3)
C3D-C2D-C7D-N1D	-179.8(2)
C1D-C2D-C7D-C6D	177.9(2)
C3E-C2E-C7E-N1E	179.9(2)
C1E-C2E-C7E-N1E	-2.1(4)
C3E-C2E-C7E-C6E	-1.3(3)
C1E-C2E-C3E-C4E	-178.0(2)
C1E-C2E-C7E-C6E	176.7(2)
C7E-C2E-C3E-C4E	0.1(4)
C1F-C2F-C7F-C6F	179.9(2)
C3F-C2F-C7F-C6F	0.8(4)
C3F-C2F-C7F-N1F	178.7(2)
C1F-C2F-C3F-C4F	-178.4(2)
C7F-C2F-C3F-C4F	0.8(4)
C1F-C2F-C7F-N1F	-2.1(4)
C2A-C3A-C4A-C5A	-0.5(5)
C2B-C3B-C4B-C5B	-0.1(4)
C2C-C3C-C4C-C5C	-0.5(4)
C2D-C3D-C4D-C5D	1.3(5)
C2E-C3E-C4E-C5E	1.1(4)
C2F-C3F-C4F-C5F	-1.3(4)
C3A-C4A-C5A-C6A	2.2(5)
C3B-C4B-C5B-C6B	0.7(4)
C3C-C4C-C5C-C6C	0.5(4)
C3D-C4D-C5D-C6D	-1.6(5)
C3E-C4E-C5E-C6E	-1.1(4)
C3F-C4F-C5F-C6F	0.2(4)
C4A-C5A-C6A-C7A	-1.9(5)
C4B-C5B-C6B-C7B	0.2(4)
C4C-C5C-C6C-C7C	0.2(4)
C4D-C5D-C6D-C7D	0.1(5)
C4E-C5E-C6E-C7E	-0.2(4)
C4F-C5F-C6F-C7F	1.4(4)
C5A-C6A-C7A-C2A	-0.2(4)
C5A-C6A-C7A-N1A	-177.9(3)
C5B-C6B-C7B-C2B	-1.7(4)
C5B-C6B-C7B-N1B	176.2(2)
C5C-C6C-C7C-N1C	176.0(2)
C5C-C6C-C7C-C2C	-0.8(4)
C5D-C6D-C7D-C2D	1.7(4)
C5D-C6D-C7D-N1D	179.5(3)
C5E-C6E-C7E-C2E	1.4(4)
C5E-C6E-C7E-N1E	-179.9(2)
C5F-C6F-C7F-N1F	-179.7(3)

C5F-C6F-C7F-C2F	-1.8(4)
C13A -C8A-C9A-C10A	0.0(4)
C9A-C8A-C13A -C12A	-0.4(4)
N1A-C8A-C9A-C10A	-174.9(3)
N1A-C8A-C13A -C12A	174.7(3)
C13B -C8B-C9B-C10B	0.2(4)
N1B-C8B-C9B-C10B	178.3(3)
C9B-C8B-C13B -C12B	1.4(4)
N1B-C8B-C13B -C12B	-176.8(3)
N1C-C8C-C9C-C10C	175.6(3)
C13C -C8C-C9C-C10C	0.2(4)
C9C-C8C-C13C -C12C	0.8(4)
N1C-C8C-C13C -C12C	-175.0(3)
N1D-C8D-C9D-C10D	-179.0(2)
C13D -C8D-C9D-C10D	-0.8(4)
C9D-C8D-C13D -C12D	0.4(4)
N1D-C8D-C13D -C12D	178.5(3)
N1E-C8E-C9E-C10E	-179.7(2)
C9E-C8E-C13E -C12E	-0.3(4)
C13E -C8E-C9E-C10E	1.9(4)
N1E-C8E-C13E -C12E	-178.7(2)
N1F-C8F-C9F-C10F	176.1(2)
C13F -C8F-C9F-C10F	0.8(4)
N1F-C8F-C13F -C12F	-177.0(3)
C9F-C8F-C13F -C12F	-2.0(4)
C8A-C9A-C10A -C11A	0.2(5)
C8A-C9A-C10A -C14A	178.9(3)
C8B-C9B-C10B -C11B	-1.6(5)
C8B-C9B-C10B -C14	-174.4(13)
C8C-C9C-C10C -C11C	-0.9(4)
C8C-C9C-C10C -C14C	-176.7(3)
C8D-C9D-C10D -C1G	-176.5(3)
C8D-C9D-C10D -C11D	0.8(4)
C8E-C9E-C10E -C14E_2	-179.7(3)
C8E-C9E-C10E -C11E	-1.8(4)
C8F-C9F-C10F -C11F	1.1(4)
C8F-C9F-C10F -C14F	-179.5(3)
C9A-C10A -C11A -C12A	-0.1(5)
C14A -C10A -C11A -C12A	-178.7(3)
C9A-C10A -C14A -F1A	-37.7(5)
C9A-C10A -C14A -F2A	84.1(5)
C9A-C10A -C14A -F3A	-156.3(4)
C11A -C10A -C14A -F1A	141.0(4)
C11A -C10A -C14A -F2A	-97.3(5)
C11A -C10A -C14A -F3A	22.4(5)
C9B-C10B -C11B -C12B	1.4(5)
C14-C10B -C11B -C12B	174.0(14)
C9B-C10B -C14-F4B	108.2(16)
C9B-C10B -C14-F5B	-19(3)
C9B-C10B -C14-F6B	-132.1(16)
C11B -C10B -C14-F4B	-65(2)
C11B -C10B -C14-F5B	168.3(18)

C11B -C10B -C14-F6B	55(2)
C9C-C10C -C11C -C12C	0.8(5)
C14C -C10C -C11C -C12C	176.5(3)
C9C-C10C -C14C -F1C	-23.1(4)
C9C-C10C -C14C -F2C	96.7(3)
C9C-C10C -C14C -F3C	-144.2(3)
C11C -C10C -C14C -F1C	161.1(3)
C11C -C10C -C14C -F2C	-79.2(4)
C11C -C10C -C14C -F3C	40.0(4)
C1G-C10D -C11D -C12D	176.9(3)
C9D-C10D -C11D -C12D	-0.4(5)
C14E_2 -C10E -C11E -C12E	177.9(3)
C9E-C10E -C11E -C12E	0.1(4)
C9F-C10F -C11F -C12F	-1.6(5)
C14F -C10F -C11F -C12F	179.0(3)
C9F-C10F -C14F -F1F	178.7(3)
C9F-C10F -C14F -F2F	57.5(4)
C9F-C10F -C14F -F3F	-60.9(4)
C11F -C10F -C14F -F1F	-1.9(4)
C11F -C10F -C14F -F2F	-123.0(3)
C11F -C10F -C14F -F3F	118.5(3)
C10A -C11A -C12A -C13A	-0.3(5)
C10B -C11B -C12B -C13B	0.3(4)
C10C -C11C -C12C -C13C	0.2(5)
C10D -C11D -C12D -C13D	-0.1(5)
C10E -C11E -C12E -C13E	1.5(4)
C10F -C11F -C12F -C13F	0.3(5)
C11A -C12A -C13A -C8A	0.5(5)
C11B -C12B -C13B -C8B	-1.6(4)
C11C -C12C -C13C -C8C	-0.9(5)
C11D -C12D -C13D -C8D	0.0(4)
C11E -C12E -C13E -C8E	-1.4(4)
C11F -C12F -C13F -C8F	1.5(4)
